# Not every worm wrapped around a stick is a guinea worm: a case of *Onchocerca volvulus* mimicking *Dracunculus medinensis*

**DOI:** 10.1186/s13071-015-1004-1

**Published:** 2015-07-16

**Authors:** Eta Ngole Mbong, Gerald Etapelong Sume, Flaubert Danbe, Walter Kang Kum, Valeri Oben Mbi, André Arsène Bita Fouda, Peter Atem

**Affiliations:** Ministry of Public Health, Yaoundé, Cameroon; WHO Country Office, Yaoundé, Cameroon; Building Bridges Cameroon (BBCAM), Yaoundé, Cameroon; P.O Box 547, Buea, Cameroon

**Keywords:** Atypical presentation, *Onchocerca volvulus*, Guinea worm, Cameroon

## Abstract

**Background:**

Despite being certified guinea worm free in 2007, Cameroon continues surveillance efforts to ensure rapid verification of any suspected reoccurrence. This includes the investigation of every rumor and confirmation of each suspicious expulsed worm. This paper presents fieldwork carried out to investigate a guinea worm rumor in Cameroon which turned out to be an *Onchocerca volvulus* mimicking *Dracunculus medinensis*.

**Methods:**

The investigation included a field visit to the subsistence farming community where the rumor was reported. During the visit, interviews were conducted with health staff who managed the case and the elderly farmer from whom the worm was retrieved. An investigation of any potential missed guinea worm cases was also conducted through interviews with community residents and reviews of the health facility’s medical records. This was combined with laboratory analyses of water samples from the community’s water sources and the retrieved worm which was removed from the patient via wrapping it around a stick.

**Results:**

Microscopy and molecular analyses of the retrieved worm revealed a female *Onchocerca volvulus* whose expulsion strongly mimicked guinea worm. In addition to presenting findings of our investigation, this paper discusses distinguishing elements between the two parasites and gives an overview of guinea worm eradication efforts in Cameroon as well as current challenges to the worm’s eradication globally.

**Conclusions:**

The investigation findings suggest the evolving *Onchocerca volvulu*s worm tropisms’ adaptive survival behavior worth further investigation. Strategies used to successfully control guinea worm in Cameroon could be adapted for *Onchocerca volvulus* control.

## Background

Guinea worm is one of the longest historically documented human parasites. Tales of the parasite are recorded in accounts penned by Greek chroniclers [1] as far back as the 2nd century BC, as well as in Egyptian medical Ebers Papyrus, dating from 1550 BC [2, 3]. Despite this longstanding knowledge about the causative agent, guinea worm disease (dracunculiasis), just as river blindness (onchocerciasis), still lingers as a neglected tropical disease associated with substantial morbidity as well as social and economic loss in already resource-poor communities and households. Unlike the latter, the former is a preventable water-borne disease affecting rural areas of countries with challenges ensuring universal access to safe drinking water. Caused by *Dracunculus medinensis,* a long and slender roundworm, the disease manifests as a nodular dermatosis resulting from the development of the parasite in subcutaneous tissues. The parasite enters a host through ingestion of stagnant water contaminated with water fleas that are infested with the worm’s larvae. Approximately a year after infection, the disease presents with a painful, burning sensation as the female worm forms a blister, usually on the lower limb. This causes temporary incapacitation (and at its worst permanent disability) resulting in loss of income (the reason the disease is termed the “disease of the empty granary” amongst the Dogon people of Mali [4]) and in children, reduced school attendance. Due to the fact that *D. medinensis* has no animal or environmental reservoir, the parasite must pass from one host (human) to another each year to survive.

Inspired by the successful eradication of smallpox in 1980, dracunculiasis and poliomyelitis were adopted in 1986 by the World Health Organization (WHO)’s policymaking body, the World Health Assembly [5], for eradication. Eradication of dracunculiasis was also formulated as a key outcome indicator of the success of the United Nations 1981–1990 International Drinking Water Supply and Sanitation Decade (IDWSSD) [6]. More than two decades after this target was set, the disease still lingers, underscoring the daunting challenge of disease control, as has been the case of the failure of previous attempts to eradicate diseases like malaria, hookworm and yaws [5].

However there is hope of eradication as the number of cases reported have markedly declined, from an estimated 3.5 million cases in 20 African and Asian countries in 1986 [5] to 126 cases in 4 countries in 2014: South Sudan, Chad, Mali and Ethiopia [7]. This decrease is driven by a decline in the number of cases reported by once-endemic and current endemic countries especially South Sudan which currently harbors about half of all reported cases worldwide [7].

The Carter Center, a leader in guinea worm’s eradication efforts, has predicted that guinea worm disease “will be the first parasitic disease to be eradicated and the first disease to be eradicated without the use of vaccines or medical treatment” [8]. Key activities being employed and which have had proven success in rolling back the disease include disease surveillance (including case management and containment), health education for behavior change, vector control, ensuring access to safe drinking-water as well as certification of eradication of once-endemic countries [9].

After reporting its first case in 1967, Cameroon with the support of international partners put in place mechanisms to control the disease. In 1997, the last confirmed case was reported and in 2007 the country was certified guinea worm free [8, 10, 11]. Knowing that eradication cannot come with complacency, surveillance efforts continue (Table 1) with a focus on areas with previous reported cases and health districts which border Chad, a neighboring country which still reports cases. Important aspects of post-certification disease surveillance include rumor investigation and laboratory confirmation of every suspicious expulsed worm. This paper presents fieldwork carried out to investigate a post-certification guinea worm rumor in Cameroon which turned out to be an *Onchocerca volvulus* mimicking *Dracunculus medinensis*. Distinguishing features between guinea worm and *Onchocerca volvulus* worm infection and control, questions for further research on adult *Onchocerca volvulu*s worm tropisms and adaptive survival behavior, and an overview of guinea worm control efforts in Cameroon as well as current challenges towards the guinea worm eradication globally, are presented and discussed in this paper.

**Table 1:** A summary of post-guinea worm eradication certification efforts in Cameroon

○ Training of community health volunteers
○ Notification and timely investigation of GW rumors,^a^ and laboratory confirmation of every expulsed worm.
○ GW case surveillance:
○ active surveillance in health districts and communities which reported cases in the past with particular attention to those bordering with Nigeria and Chad
○ infection transmission interruption and case containment measures
○ obligatory weekly reporting by all health districts in the country as part of the National integrated epidemic-prone disease surveillance and response system (IDSR)
○ Information, education and communication on GW disease including vulgarization of information on the monetary reward
○ Money reward system in case of confirmed rumor
○ 4000 francs CFA (6.7 US dollars^b^) for the person who identified and declared a case
○ 23,000 francs CFA (38.5 USD) reward given to an indigenous patient with GW ·
○ 3000 FCFA (5.0 USD) for an imported patient
○ 10,000 FCFA (16.7 USD) for the Health facility which manages the case
○ 40,0000 (67 USD) for the patient’s community to support the treatment of water sources

^a^42 rumors in all have been reported and investigated since 2009. But for 2009 which registered 5 rumors of which just 3 (60 %) were investigated within 24 h, all other rumors reported during the years that followed were investigated within 24 h

^b^1 USD = 597.434 FCFA (local currency)

## Methods

### Site investigated

The rumor was reported in Lala-Mission (GPRS coordinates: 04, 79953 North and 009, 77902 East), a peasant community in the Littoral Region of Cameroon, with an estimated total population of 741 inhabitants. Though a non-endemic village, prior to guinea worm eradication certification of Cameroon, two previous guinea worm rumors had been registered in this community (in 2007 and 2008).

### Case investigation

#### Team and procedure

The investigation team was made up of epidemiologists and laboratory staff of the District and Regional Health Services where the case had been reported in collaboration with the Disease Control Department of the Ministry of Health (MoH). After a courtesy visit to local administrative authorities and community leaders, the investigation began with a field visit to the Integrated Health Center where the case had been reported followed by a visit to the patient’s home.

At the health center, staff were interviewed on how the case was contained and managed and the patient’s records reviewed along as well as outpatient registers and files of other patients in search of cases which may have presented with similar symptoms but were missed or unreported by the facility. The worm sample retrieved from the patient and conserved in formalin was collected by the investigation team for analyses.

The patient had been discharged from hospital after case containment and worm retrieval. At the patient’s home an in-depth interview of the patient was conducted, facilitated by the patient’s daughter who first suspected the case. The interview ruled out the patient having come into contact with water sources after the emergence of the worm and also focused on the patients’ travel history. Physical examination of the patient revealed a partially healed wound with no nearby skin opening and no evidence of ocular, nodular or other skin presentation of onchocerciasis. The home visit was followed by an active search for missed or unreported cases of the disease in the community through interviews of the patient’s neighbors and other community members. Thereafter the water sources of the community were mapped out and water samples collected including those used by the patient for drinking and domestic chores.

#### Case presentation and management

**Fig. 1:**
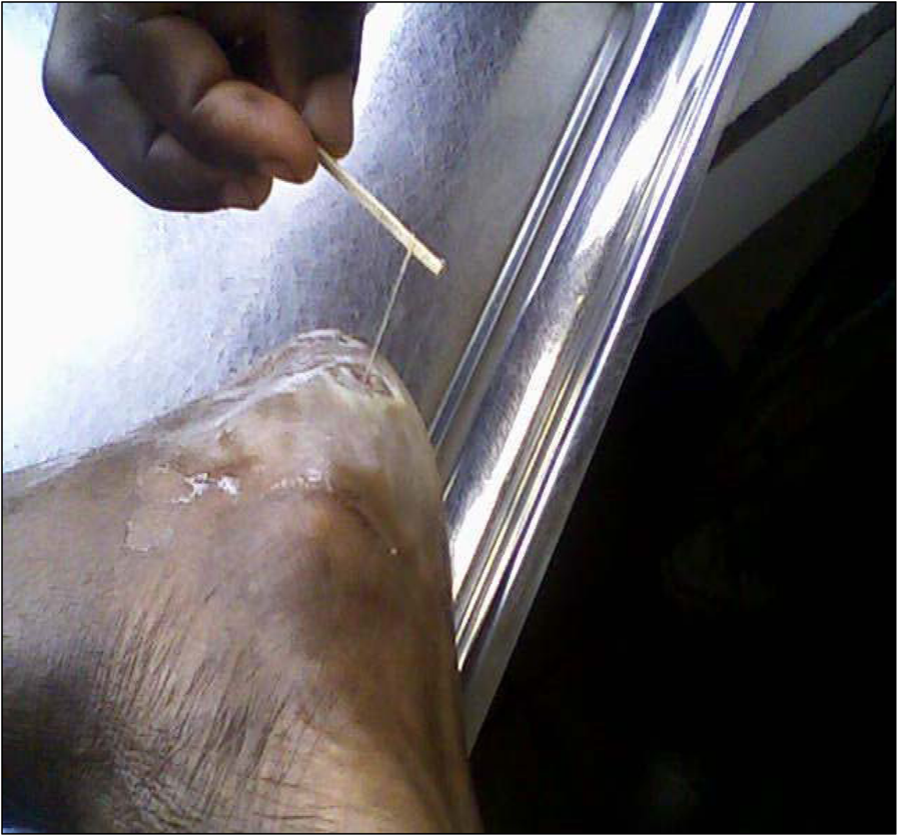
Worm being extracted using wrapping-around-a-stick technique (Lala-Kola village, Littoral Region of Cameroon, March 2009)

**Fig. 2:**
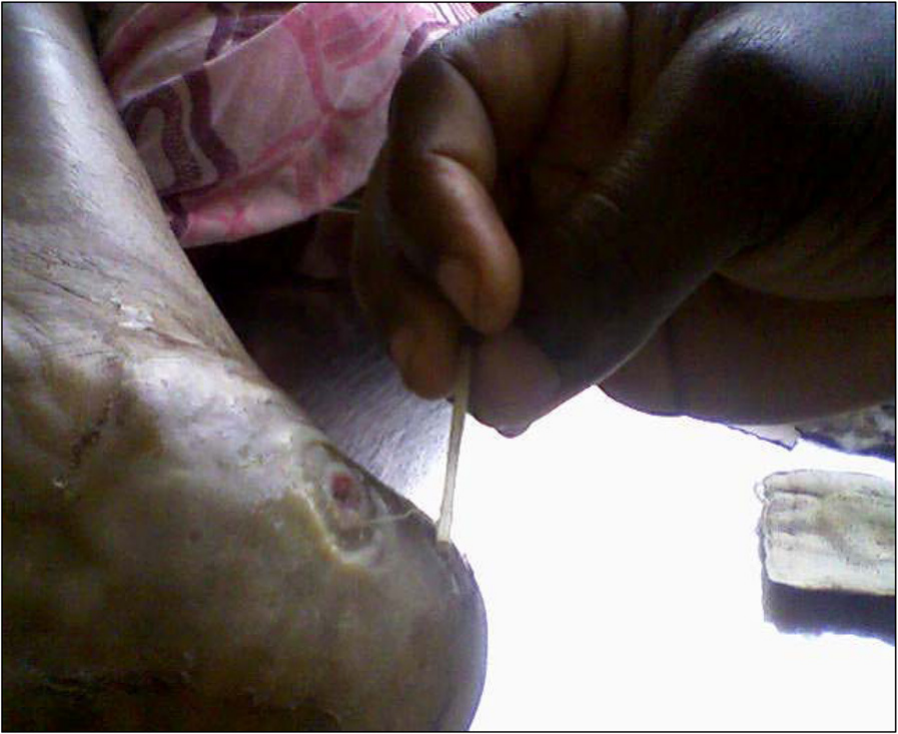
Heel lesion and worm extirpation around a stick Lala-Kola village (Littoral Region of Cameroon, March 2009)

The worm was retrieved from an elderly woman (77 years old). She was a subsistence farmer with first-time presentation of expulsion of a worm from a limb and no other known co-morbidities. She had been received at the Lala-Mission Health Center in February (during the dry season), three weeks prior to the arrival of the investigation team. Two weeks before seeking medical attention, she complained of itches around her right heel which later swelled, became tender and papular, and progressively ulcerated without blistering (refer to Figs. 1 and 2). The patient’s daughter, a community health volunteer with some knowledge about guinea worm given the prior rumors of 2007 and 2008, on taking a closer look noticed the free end of a worm hanging out of the wound.

After two failed attempts at home by her daughter to retrieve the worm by wrapping around a stick which according to her daughter’s account sectioned the worm by about 5 cm, the patient was taken to the Health Center. By the third day of case containment the entire worm (65 cm by 5 mm) was retrieved by wrapping it around a stick, conserved in formalin and the case notified to district health authorities.

#### Visit and collection of samples from the village and patient’s water sources

**Fig. 3:**
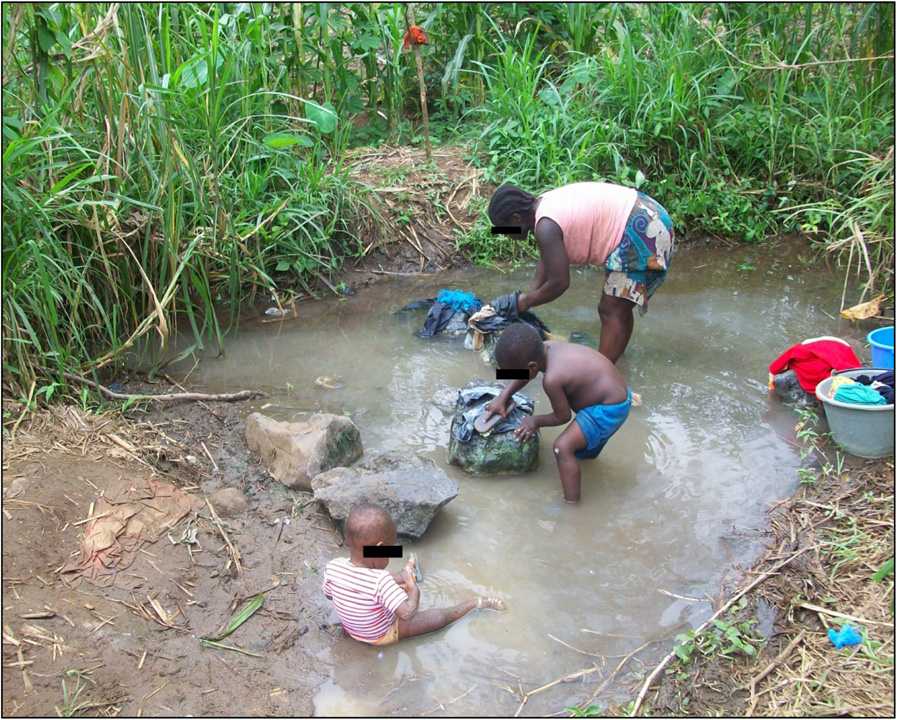
Pond being used for laundry by a Lala-Mission mother and her kids (Littoral Region of Cameroon, March 2009)

Led by community representatives, the village’s water sources were mapped out. The community’s main water sources consisted of two rivers (Ntam I and Dibombe), a stream (Ntam II) and a water pond (locally referred to as “Duck’s pond”), which due to the absence of pipe-borne water, are used for drinking, bathing and domestic chores (Fig. 3). The patient acknowledged using these water sources without filtering for drinking and domestic chores as well as another stagnant pond found on the way to her farm. Two water samples of 5-litres each were collected from each of these water sources, one in the morning and the other in the evening, at peaks when most residents of the community came to fetch water. The samples were cloth filtered and examined for the presence of cyclops and guinea worm larvae as described in the section on laboratory analyses below.

#### Patient’s travel history prior to the investigation

Inquiry about the patient’s travel history revealed that the patient rarely traveled. She had made a trip two years prior to the emergence of the worm to some nearby towns not known in the past to be guinea worm-endemic. She reported no travel during the year when the worm was expulsed.

#### Worm sample collection and education on preventive measures

At the end of the mission, the investigation team left with a partially sectioned 65 cm long by 5 mm wide worm and water samples. The mode of exteriorization of the worm, its length (almost thrice the average length of an adult onchocerca worm), the village’s sources of water and the past guinea worm rumors reported by the village suggested a likely case of guinea worm which the community was reassured would be confirmed by analyses of the worm and water samples. Prior to the team’s departure, health education sessions on the disease and preventive measures were organized within the community with the support of the local health authorities including calls to remain vigilant.

### Ethical considerations

The investigation mission was authorized by authorities of the Ministry of Public Health, and review of patient medical records, by authorities of the health facilities visited. Informed verbal consent was obtained from those interviewed and data obtained during the investigation were treated with confidentiality.

## Results

### Water sample analyses findings

Collected water samples were filtered through two sets of 8-inch sieves in succession. Thereafter, retained cyclops were washed off the finer mesh into 10 ml specimen bottles containing 2.5 ml of distilled water. These were kept in the refrigerator for approximately 24 h and later emptied into a cross hatched petri-dish and counted under a dissecting microscope with a Talley hand counter. After this initial census, retrieved cyclops were pipetted onto a glass slide, cover-slipped and examined under the compound microscope for the presence of guinea worm larvae. No infected cyclops were observed.

### Worm analyses findings

The formalin preserved worm sample was sent for analyses to the WHO Collaborating Center for Research, Training and Eradication of Dracunculiasis based in the Center for Disease Control and Prevention (CDC) Atlanta, USA with the support of the WHO Cameroon country office. Microscopy surprisingly revealed an un-encapsulated adult worm with a rough cuticle made up of external ridges that ran around the body and internal bars/band striae (two striae per ridge, one under adjacent ridges and one between adjacent ridges) characteristic of *O. volvolus*. This, the laboratory report noted, is unlike the cuticle of *D. medinensis* which on microscopy is smooth (devoid of external ridges or internal striae) and thicker. The specimen also had few microfilariae in utero suggesting it was a female worm. 18S rRNA gene sequence analysis, a method that differentiates *D. medinenis* from other nematods [12], further ruled out guinea worm and confirmed *O. volvolus*.

## Discussion

This paper describes an atypical presentation of *Onchocerca volvulus*: exteriorization of a 65 cm (unusually long for an onchocerca) adult worm, through the heel of an elderly female subsistence-farmer with no nodular, eye or other skin presentation of onchocerciasis or co-morbidities. This is one of the few documented instances of *Onchocerca volvulus* strongly mimicking guinea worm being extracted from the sole of the foot [10, 13]. Other sites have been from the chin, chest and hip [13].

Another striking feature was the presence of the adult *O. volvolus* worm free (un-encapsulated) in the tissues of the sub-dermis an observation which Eberhard and collaborators [13] reported had been observed in all other cases of *O. volvulus* mimicking guinea worm they had come across. This is unlike the characteristic feature of human infection with *O. volvulus* whereby adult worms are encapsulated in subcutaneous fibrous tissue, an onchocercoma (nodule) [14] which harbors coiled adult worms. The parasite was first reported and described in 1893 by Leuckart from such nodules under the skin of a patient in Ghana [14]. Nodules may also occur in deep tissues and not be readily evident or palpable [15].

Adult worms not encased in fibrous nodules have long been sources of speculation. Mohammed [16] speculated that nodule formation was primarily a response to reaction around degenerating or injured adult worms which may explain why nodules are often identified over bony prominences. This theory however does not explain why viable young worms have also been observed in some nodules. The worm specimen extirpated from the elderly female patient contained microfilariae indicating that full maturation and mating had occurred, yet without the formation of a nodule. Information garnered from interviews with health center staff who extirpated the worm did not suggest the worm was initially contained within a nodule and accidentally removed during the course of extraction. Other cases of *O. volvulus* mimicking guinea worm in literature have not suggested this either [13]. Though this case, as well as similar cases reported in literature have been from areas in which mass Ivermectin drug administration program for river blindness control was carried out, there is no evidence that Ivermectin interferes with nodule formation and accounted for the absence of a nodule [10]. This however merits further investigation.

The extracted worm was an adult female, an observation which has been noticed with all other *O. volvulus* worm cases whose expulsion mimicked guinea worm [13]. This may suggest that female *O. volvulus* just as female guinea worms have or may be acquiring tropism for body surfaces and extremities where they secrete enzymatic substances which facilitate their expulsion to the exterior in order to propagate species. This needs further investigation. Prior to expulsion, a classic guinea worm pre-expulsion blister was not formed and the patient’s limb not as inflamed as would be expected with guinea worm disease especially after the failed attempts at its extirpation at home prior to seeking medical assistance (refer to Figs. 1 and 2).

The worm sample was thinner than would be expected for a guinea worm (refer to Fig. 1) though cases have been reported of thin guinea worms being expulsed, whereby the uterine tubes, rather than the entire body of the worm, protrudes out of the lesion, creating the appearance of a much thinner worm [13, 17]. Size in a clinical setting being a subjective measure, further microscopic examination is always necessary.

People living in agricultural zones especially farmers as was the case of the patient who was the subject of this investigation, are known to have higher susceptibility to onchocerciasis given the long hours they spend outdoors where they are exposed to bites of the blackfly, the onchocerca vector [18]. Cases of complications of the disease including blindness, lizard skin and nodules are common in such communities. Rapid flowing rivers as well as stagnant ponds used for drinking water, both of which were observed in the patient’s community, are respectively risk factors for onchocerciasis and guinea worm disease. We are uncertain if niches with a mix of rapid flowing rivers, stagnant water and agricultural relief as was the case of the village under investigation favor the occurrence not only of either of the diseases but also cases of Onchocerciasis mimicking guinea worm.

The patient had received for several consecutive years Ivermectin chemotherapy, an important component of river blindness control efforts in high onchocerca-burden areas like Cameroon. Are female *O. volvulus* gaining adaptive cutaneous and extremities tropisms to propagate species in response to mass Ivermectin distribution control efforts? These are questions worth investigating whose answers may explain why more and more, *O. volvulus* mimic guinea worm expulsion [13]. These forms of *O. volvulus* may have been common in the past in guinea worm endemic areas and mistaken for guinea worms during attempts to systematically identify all cases of the disease as part of eradication efforts, but are now distinguished from the latter with the decline of guinea worm disease cases.

Though dracunculiasis and onchocerciasis are both diseases whose geographical distribution is limited to tropical and subtropical areas, the control and eradication of guinea worm disease, unlike onchocerciasis, is more likely as its transmission is seasonal [19], diagnosis is unambiguous by visual recognition of an emerging worm through a painful blister or ulcer, the intermediate host is non-airborne and there is no known animal reservoir [20]. Moreover, there is no further multiplication of the parasite in the vector, unlike other parasitic diseases. This potential for eradication was asserted in 1993 by the International Task Force for Disease Eradication, which after reviewing 94 infectious diseases, concluded that guinea worm is one of six eradicable diseases [21].

**Fig. 4:**
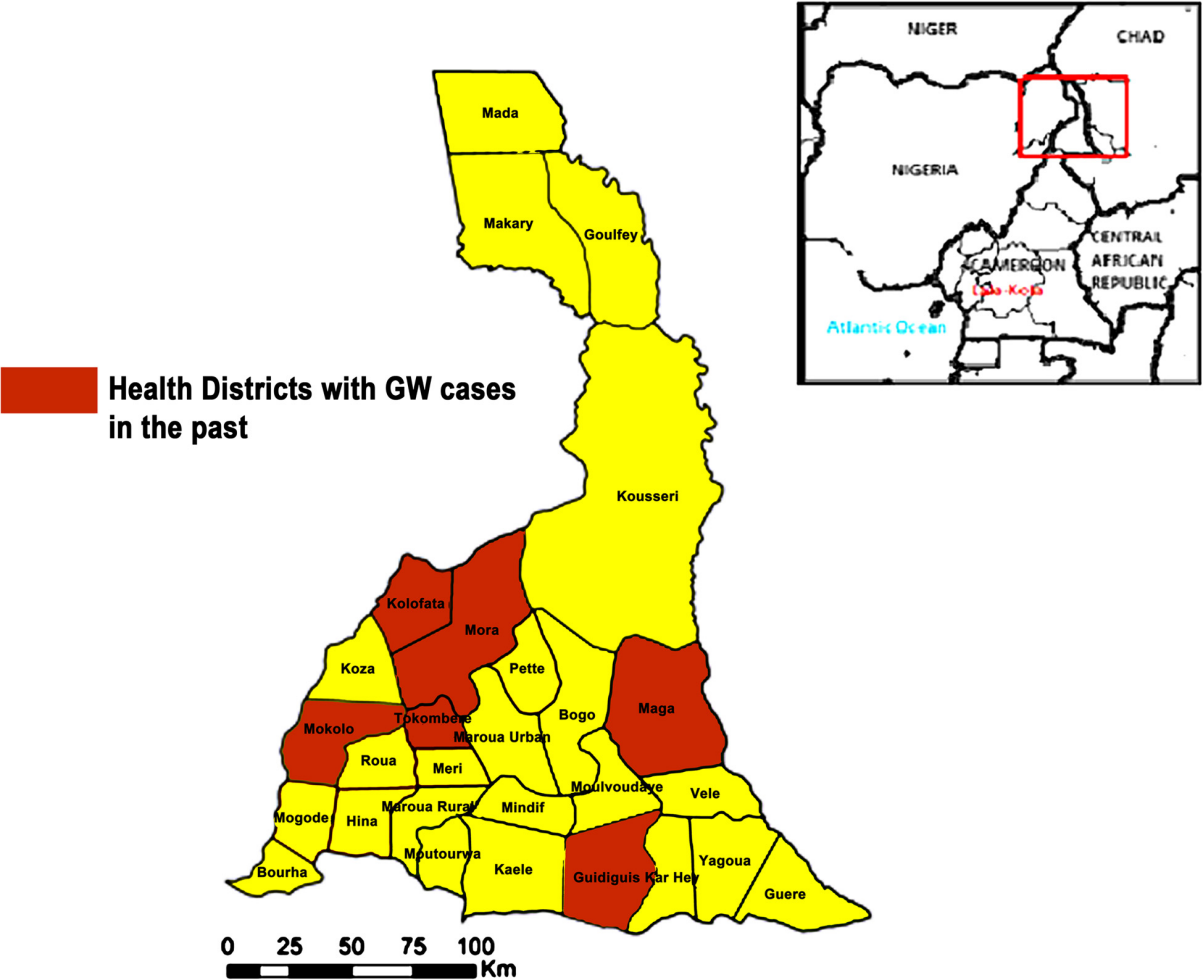
Health districts in Cameroon which reported guinea worm cases pre-eradication-certification

Like all previous guinea worm endemic countries, Cameroon has experienced three stages of control of the disease: the endemic phase prior to the 1980s, the precertification phase (1997–2006) and the post-certification phase (2007 onwards). Surveillance, like in all disease programmes, plays a key role in guinea worm control in Cameroon. During the endemic phase and two years prior to the set-up of the national guinea worm control programme in 1990, national case searches were started for more accurate reporting of the annual incidence [9]. Active surveillance was carried out in health districts which reported cases as well as at risk districts (Fig. 4). This included house-to-house case searches within communities with the aid of guinea worm photo identification card to assess whether anyone had seen a person with an emerging worm. Community-based surveillance (CBS) for detecting, containing and reporting cases was also put in place which relied on community health volunteers, especially for hard to reach endemic and at-risk communities with limited access to primary healthcare services, supported by supervisors who trained, monitored and collected monthly reports from volunteers. Suspected cases of dracunculiasis were reported for prompt investigation and containment, and cases were reported monthly to the national level (including zero cases).

With the support of partners such as WHO, UNICEF, the Japanese International Cooperation Agency (JICA) and the Carter Center and in addition to the above described surveillance activities, initiatives were put in place to interrupt worm transmission and ensure proper case containment, two mainstays of guinea worm control programmes [9]. Due to the absence of a known effective drug against the parasite, the development of drug resistance unlike as is the case of Onchocerciasis control, was not a concern. Aside the validation within 7 days by a guinea worm supervisor of the suspected case presented in this paper, all four other recommendations for guinea worm containment [22] were followed by the health facility during the management of the case.

Unlike onchocerciasis control, guinea worm control in Cameroon partners with the Integrated Disease Surveillance and Response (IDSR) system, which provides a nationwide opportunity to improve dracunculiasis surveillance, especially in formerly endemic and non-endemic areas where active dracunculiasis-specific surveillance is not in place. To improve reporting as well as overcome pitfalls associated with facility-based IDSR reporting, especially as the number of cases declined during the precertification phase, a monetary reward system for notified cases was instituted, a strategy recommended by the International Commission for the Certification of Dracunculiasis [23] and employed during the smallpox eradication campaign [24]. The programme also relies on other disease control programmes and initiatives such as the polio surveillance network, national immunization days and mass preventive chemotherapy programmes for large-scale house-to-house case search activities.

The national guinea worm control programme operates at the national level through its provision of overall strategic guidance, regular monitoring and evaluation. All interventions are implemented and managed by the national primary healthcare system with outreach services under the supervision of district health authorities. Where healthcare infrastructure is severely limited, CBS networks directed at endemic or at-risk communities were managed directly by a secretariat at the national level with varying levels of interaction and liaison with district health services. As the number of cases declined during the precertification phase, this responsibility was transferred to the district’s primary healthcare system. The *Onchocerca volvolus* control programme started 5 years after the national guinea worm control programme [25] as part of the African Programme for Onchocerca Control (APOC)’s could learn from relevant guinea worm eradication efforts described above as well as those of other disease control programmes in Cameroon.

Almost a decade after being certified guinea worm free, post certification surveillance continues. This is a crucial element in the final stage of the dracunculiasis eradication programme until the last case worldwide is eradicated. This includes the laboratory confirmation of every suspicious worm, as was done for the case presented in this paper. Particular attention is given to areas which reported cases in the past most of which are located in the North of Cameroon (Fig. 4) as well as areas close to Chad, a neighboring country which after 10 years of not having reported an indigenous case started reporting guinea worm cases [9, 26, 27] including confirmed cases of *Dracunculus medinensis* in dogs in 2010 [28]. This underscores the role of sustained surveillance in guinea worm control, especially when the disease burden is reduced or transmission interrupted [9]. Unlike Chad, cases of guinea worm in dogs have not been reported in Cameroon. However given the long borders Cameroon shares with Chad, it is an opportunity, to include veterinarians in Cameroon in post-certification community-based controls efforts in a One Health approach.

One may be tempted to think that the late start of a guinea worm control program and national case searches in Chad compared to Cameroon (1993 in the former compared to 1988 in the latter) could explain why the disease still persists in Chad [9]. This does not explain why neighboring Nigeria which started national case searches and initiated a CBS at the same time as Cameroon was certified guinea worm free 6 years later (2013) than Cameroon [22]. Determinants of how long countries take to interrupt transmission and achieve zero reported indigenous cases include the burden of the disease, transmission dynamic complexities at the individual and community level, country-specific operational implementation of control programs and the intensity and accuracy of control interventions [9]. Sustained political commitment, sustained surveillance when the disease burden is reduced or transmission interrupted, sustained programme effectiveness and reporting, and integrating control into the primary health care system are lessons which have been learned and shown to improve guinea worm control as well as progress towards eradication [9]. These are also lessons from which onchocerciasis control programmes could learn from.

Although the 1991 and 2004 World Health Assembly goals to eradicate dracunculiasis globally in 1995 and 2009, respectively, are yet to be achieved [9, 29], 197 countries, areas and territories have been certified guinea worm free, leaving just 9 still to be certified: 4 endemic countries (South Sudan, Chad, Mali and Ethiopia), three countries in the precertification stage (Ghana, Kenya and Sudan) and two countries never known to have had endemic guinea worm (Angola and the Democratic Republic of Congo) [22].

Failures in surveillance and containment, lack of clean drinking water, conflict and insecurity in Mali and South Sudan, movement of people from place to place (nomadic populations, cattle herders and persons displaced due to conflicts), and an unusual epidemiologic pattern in Chad are the main current challenges to dracunculiasis eradication [9, 22, 28]. Movement of internally displaced persons, as well as refugees into the once-endemic North of Cameroon (as well as into Nigeria), from guinea worm endemic neighboring Chad due to the ongoing Boko Haram military conflict calls for the reinforcement of post-certification surveillance activities in Cameroon as well as Nigeria.

Microscopic morphological examination of the cuticle can readily distinguish *Onchocerca* worms from *Dracunculus* [13], a rapid and low-tech procedure laboratory field workers in endemic and once-endemic zones engaged in disease surveillance would benefit from training in. This will ensure their knowledge that not all worms extracted from the skin even by wrapping around a stick are guinea worms.

## Conclusion

This paper is a report of field work carried out in a guinea worm eradication certified country, Cameroon. The site of the study was a subsistence-farming community where a team was deployed to investigate a guinea worm rumor which on laboratory analyses of worm samples turned out to be an *Onchocerca volvulus* mimicking *Dracunculus medinensis* and presenting atypically through heel expulsion. Changing adult *Onchocerca volvulu*s worm tropisms and adaptive survival behavior worth investigating, probably influenced by factors such as mass Ivermectin chemotherapy and geographic niches, may explain this atypical presentation. Onchocerca volvulus control in Cameroon could learn from relevant guinea worm control strategies, which control efforts, need to be sustained.
